# Association of Residency Training With Metabolic Measures

**DOI:** 10.1001/jamanetworkopen.2020.5120

**Published:** 2020-04-30

**Authors:** Takahiro Matsuo, Gautam A. Deshpande, Hiroko Arioka, Daiki Kobayashi

**Affiliations:** 1 Department of Infectious Diseases, St Luke’s International Hospital, Tokyo, Japan; 2 Department of General Internal Medicine, St Luke’s International Hospital, Tokyo, Japan; 3 Department of General Medicine, Juntendo University, Tokyo, Japan; 4 Graduate School of Public Health, St Luke’s International University, Tokyo, Japan; 5 Department of Community-Based Medicine, Fujita Health University, Toyoake, Japan

## Abstract

This cohort study investigates body mass index, body fat percentage, and laboratory measures of lifestyle-related clinical parameters in a population of medical residents compared with matched non–health care practitioner controls.

## Introduction

The rigors of residency training may require physicians to undergo substantial, and often dramatic, changes in lifestyle. Residents can be at high risk of having unhealthy habits, including obtaining less physical activity and sleep^1^ and experiencing high levels of stress.^2^ Little is known about the clinical ramifications of these changes on body mass index, body fat percentage, and other laboratory measures. The aim of this cohort study is to investigate the associations among these parameters in a population of medical residents compared with matched non–health care practitioner controls.

## Methods

This study was approved by the institutional review board at St. Luke’s International Hospital in Tokyo, Japan, with a waiver of informed consent granted because the study used no individual identifiers and posed minimal risk to participants. This study follows the Strengthening the Reporting of Observational Studies in Epidemiology (STROBE) reporting guideline.

We conducted a matched cohort study of all first-year postgraduate residents between 2004 and 2015 at a large academic tertiary care medical center and analyzed the data in January 2017. Data for each resident were matched by age, sex, and body mass index with 2 non–health care practitioner controls from a community health check-up program at St. Luke’s International Hospital, Center for Preventive Medicine, Tokyo, Japan.

The primary outcome was difference in lifestyle-related clinical parameters, including body mass index, body fat percentage, hypertension, diabetes, and lipid profile, between baseline and 1-year follow-up. Secondary outcomes, including changes in length of sleep, frequency of exercise, alcohol consumption, and smoking, were investigated using a self-reported questionnaire.

We first compared baseline characteristics between residents and controls by using *t* tests and χ^2^ tests as appropriate. We then applied similar tests to evaluate differences in health-related measurements between at baseline vs at 1-year follow-up. Variables demonstrating statistical significance on univariate analyses, or those considered clinically important, were included in multivariable analyses using a mixed-effect model. All analyses were performed using Stata statistical software version 11 (StataCorp) with statistical significance set at 2-sided *P* < .05.

## Results

In total, 281 residents were compared with 562 controls (573 men [68.1%]). The mean (SD) age of all participants was 25.5 (1.9) years, and the mean (SD) body mass index (calculated as weight in kilograms divided by height in meters squared) was 21.3 (2.6) at baseline. Baseline characteristics of residents and controls are shown in Table 1. The most striking differences between residents and controls were current smoking rates (2 residents [0.7%] vs 160 controls [28.5%]), the number reporting insufficient sleep (69 residents [25.0%] vs 254 controls [45.7%]), and the number reporting daily alcohol use (52 residents [18.5%] vs 192 controls [34.2%]).

**Table 1.** Baseline Characteristics of Residents and Non–Health Care Practitioner Controls

Characteristic	Value, Mean (SD)		*P* value
	Residents (n = 281)	Controls (n = 562)	
Male, No. (%)	191 (68.0)	382 (68.0)	>.99
Age, y	25.5 (1.9)	25.5 (1.9)	>.99
Height, cm	169.0 (8.7)	168.0 (8.0)	.93
Weight, kg	61.2 (10.8)	60.5 (10.3)	.81
Body mass index	21.3 (2.6)	21.3 (2.6)	>.99
Body fat, %	19.2 (4.7)	20.4 (5.2)	.001
White blood cell count, × 10^9^/L	5.9 (1.5)	5.7 (1.4)	.96
Hemoglobin, g/dL	14.2 (1.4)	14.3 (1.3)	.07
Total protein, g/dL	7.3 (0.4)	7.2 (0.3)	.92
Albumin, g/dL	4.6 (0.3)	4.6 (0.2)	.36
Total bilirubin, mg/dL	0.9 (0.4)	0.9 (0.4)	.91
Aspartate aminotransferase, IU/L	23.8 (62.9)	19.7 (6.2)	.93
Alanine aminotransferase, IU/L	18.9 (22.4)	21.1 (15.7)	.05
Gamma-glutamyl transpeptidase, IU/L	22.0 (18.3)	25.1 (24.3)	.03
Alkaline phosphatase, IU/L	182.0 (52.1)	185.8 (56.9)	.19
Total cholesterol, mg/dL	172.7 (26.4)	173.1 (28.2)	.42
High-density lipoprotein, mg/dL	66.6 (14.1)	59.4 (13.6)	>.99
Low-density lipoprotein, mg/dL	90.9 (24.3)	98.3 (26.7)	.001
Triglycerides, mg/dL	56.8 (26.4)	72.6 (43.4)	.001
Uric acid, mg/dL	5.4 (1.3)	5.4 (1.3)	.53
Glycated hemoglobin, %	5.2 (0.3)	5.2 (0.3)	.63
Creatinine, mg/dL	0.8 (0.2)	0.8 (0.1)	.49
Systolic blood pressure, mm Hg	119 (14.2)	112.7 (13.1)	>.99
Diastolic blood pressure, mm Hg	66.8 (8.5)	70.2 (9.3)	>.99
Current smoking, No. (%)	2 (0.7)	160 (28.5)	.001
Insufficient sleep, No. (%)	69 (25.0)	254 (45.7)	.001
Alcohol, No. (%)			
Rarely	212 (75.4)	311 (55.3)	.001
Occasional	17 (6.1)	59 (10.5)	
Nearly daily	52 (18.5)	192 (34.2)	
Exercise, times/wk, No. (%)			
Seldom	114 (40.6)	266 (47.3)	.001
1-2	128 (45.6)	210 (37.4)	
3-5	29 (10.3)	49 (8.7)	
Nearly daily	10 (3.6)	37 (6.6)	

SI conversion factors: To convert albumin to g/L, multiply by 10.0; alanine aminotransferase to μkat/L, multiply by 0.0167; alkaline phosphatase to μkat/L, multiply by 0.0167; aspartate aminotransferase to μkat/L, multiply by 0.0167; cholesterol to mmol/L, multiply by 0.0259; creatinine to μmol/L, multiply by 88.4; gamma-glutamyl transpeptidase to μkat/L, multiply by 0.0167; hemoglobin to g/L, multiply by 10.0; glycated hemoglobin to proportion of total hemoglobin, multiply by 0.01; total bilirubin to μmol/L, multiply by 17.104; total protein to g/L, multiply by 10.0; triglycerides to mmol/L, multiply by 0.0113; uric acid to mmol/L, multiply by 0.0595.

Body mass index is calculated as weight in kilograms divided by height in meters squared.

Multivariable analyses revealed statistically significant increases in body fat percentage (mean, 0.25% per year; 95% CI, 0.03% to 0.48% per year; *P* = .03) and low-density lipoprotein cholesterol level (mean, 1.93 mg/dL per year; 95% CI, 0.36 to 3.49 mg/dL per year; *P* = .02), and decreases in high-density lipoprotein cholesterol level (mean, −3.01 mg/dL per year; 95% CI, −3.86 to −2.17 mg/dL; *P* = .001) (to convert cholesterol values to mmol/L, multiply by 0.0259) and diastolic blood pressure (−1.43 mm Hg per year; 95% CI, −2.14 to −0.71 mm Hg; *P* = .001) among residents compared with non–health care practitioners (Table 2). In addition, residents reported shorter duration of sleep (odds ratio, 2.16; 95% CI, 1.61 to 2.89) and less exercise (odds ratio, 2.69; 95% CI, 1.70 to 4.26) compared with the controls.

**Table 2.** Comparison by Multivariate Analysis of Clinical Parameters Between Physicians and Non–Health Care Practitioners at 1-Year Follow-up

Parameter	Change between residents and non–health care practitioners, adjusted mean difference (95% CI)	*P* value
Body fat, %	0.25 (0.03 to 0.48)	.03
Body mass index	0.04 (−0.06 to 0.13)	.45
Low-density lipoprotein, mg/dL	1.93 (0.36 to 3.49)	.02
High-density lipoprotein, mg/dL	−3.01 (−3.86 to −2.17)	.001
Triglycerides, mg/dL	4.1 (−3.20 to 11.3)	.27
Systolic blood pressure, mm Hg	−0.85 (−1.84 to 0.15)	.10
Diastolic blood pressure, mm Hg	−1.43 (−2.14 to −0.71)	.001

SI conversion factors: To convert cholesterol to mmol/L, multiply by 0.0259; triglycerides to mmol/L, multiply by 0.0113.

Body mass index is calculated as weight in kilograms divided by height in meters squared.

## Discussion

Deleterious changes in body fat percentage, low-density lipoprotein level, high-density lipoprotein level, diastolic blood pressure, and quantity of sleep were more frequent in first-year resident physicians than in the general population. Residents may be at higher risk for poor physical health as a result of less primary care maintenance,^3^ in addition to noncompliance with national health recommendations regarding physical activity and healthy eating practices.^4^ Insufficient self-maintenance among residents is likely associated with several factors, including excess working hours leading to physical inactivity^5^ and poor recognition of the long-term effects of unhealthy habits.^6^

This study has limitations. It was conducted in a single institution in Tokyo before work-hour reforms were instituted in 2017. Also, this study only focuses on the first year of residency.

Because deleterious changes in physicians’ lifestyle can also lead to decreased quality of patient care, well-being and self-care education provided early in residency may be an important component for young physicians’ training. Possible changes in clinical parameters past the first year of training, whether continued deterioration or potential amelioration, remain unknown and, along with intervention studies, is a needed area of future research.
